# Tripartite communication in COVID-19 infection: SARS-CoV-2 pathogenesis, gut microbiota and ACE2

**DOI:** 10.2217/fvl-2021-0231

**Published:** 2022-07-22

**Authors:** Sara Ahmadi Badi, Shohreh Khatami, Seyed Davar Siadat

**Affiliations:** ^1^Microbiology Research Center (MRC), Pasteur Institute of Iran, Tehran, 1316943551, Iran; ^2^Department of Mycobacteriology and Pulmonary Research, Pasteur Institute of Iran, Tehran, 1316943551, Iran; ^3^Biochemistry Department, Pasteur Institute of Iran, Tehran, 1316943551, Iran

**Keywords:** COVID-19, gut microbiota and ACE2, SARS-CoV-2

COVID-19 caused by a novel coronavirus, named SARS-CoV-2, is responsible for the current viral pandemic. COVID-19 infection can be accompanied by gastrointestinal (GI) complications [1]. The following factors affect the susceptibility to and severity of the COVID-19 infection: ACE2, the main entrance receptor of SARS-CoV-2 and the vital counterbalancing of the renin-angiotensin system (RAS); and gut microbiota, the diverse and dynamic microbial colonizers of the GI tract [2]. Here, we discuss the correlation of ACE2 and gut microbiota with SARS-CoV-2 pathogenesis concerning susceptibility to and severity of COVID-19.

## ACE2 functions

ACE2, a key component of RAS, is expressed on various organs, such as the respiratory and GI tracts. ACE2 exerts many protective effects on the host, including modulating fluid homeostasis, inflammatory responses and fibrosis by degrading Ang II, the accumulation of which is observed during acute lung injury and fibrosis [2]. According to previous studies, ACE2 mainly mediates the entrance of SARS-CoV-2, and the virus significantly downregulates ACE2 in the lungs, which could result in the development of COVID-19, lung failure and death [3]. As mentioned, SARS-CoV-2 may infect the GI tract due to the expression of ACE2 on the intestinal epithelial cells.

## Gut microbiota & COVID-19 infection

In addition, growing data demonstrated the critical role of gut microbiota in the severity of COVID-19 and it has been reported that gut microbiota alters following infection [4]. Gut microbiota is a complicated microbial community considered a crucial environmental factor that regulates a wide variety of host functions, especially the modulation of the metabolism and the immune system. It plays a key role in determining health and disease status [5]. Furthermore, the gut microbiota can influence the function of distant organs such as lungs, and is called the gut–lung axis. Interestingly, the lung microbiota pattern (i.e., the microbial colonizers of the respiratory tract) and the type of immunity against respiratory pathogens are affected by gut microbiota. Moreover, respiratory infection changes the composition of the gut microbiota and its executive roles [6]. Lung immunity under the regulation of gut microbiota is established by several factors, such as the interaction between microbe-associated molecular patterns and pattern recognition receptors, gut barrier function, the activity of resident immune cells in lamina propria and microbial metabolites. For example, the gut microbiota-derived metabolites, short-chain fatty acids, affect lung immunity against influenza virus by modulating the differentiation of immune cell precursor from bone marrow to lung, leading to the dampening of the main characteristics of a respiratory viral infection, including inflammation and severe tissue damage [7].

It has been reported the altered gut and lung microbiota composition in patients with COVID-19 which is characterized by enrichment of the opportunistic pathogens abundance [4]. The prominent symbionts of the gut microbiota with an anti-inflammatory effect, such as *Faecalibacterium prausnitzii*, *Eubacterium rectale, Clostridium butyricum, Clostridium leptum* and several species of *Bifidobacteria*, are depleted in COVID-19 patients in comparison with healthy controls [8,9]. The dysbiotic gut microbiota pattern is correlated with the severity of SARS-CoV-2 pathogenesis, elevated inflammatory mediators and tissue damage markers, including CRP and lactate dehydrogenase [8]. Although few studies have examined the lung microbiota composition in the COVID-19 patients, the perturbation of lung microbiota with opportunistic pathogens has been reported [10,11].

It has been noticed that the gut microbiota community is a dynamic microbial population shaped by various factors, especially diet, pre/probiotic consumption and antibiotic regimes, the consideration of which could be helpful to restore a symbiotic gut microbiota and control COVID-19 infection. Furthermore, high-risk individuals infected with SARS-CoV-2, such as diabetic, hypertensive and cancer patients, need to have complex pharmacological diets, named polypharmacy, which can influence the richness and diversity of gut microbiota and resistome (i.e., the sum of antibiotic-resistance genes). Antibiotics and nonantibiotic medication may mediate the perturbations of both gut microbiota composition and resistome. As a result, such medication (use or abuse) could be responsible for increasing the resistance of the gut microbiota members that can spread antibiotic-resistance genes in pathogens [12,13]. This condition may affect the protective role of commensal bacteria and the efficacy of medicines, such as azithromycin, used in managing the COVID-19 pandemic and the challenging eradication of opportunistic pathogens during the superinfection of COVID-19 patients [14]. Therefore, it is important to consider the impact of polypharmacy on the gut microbiota composition and resistome during COVID-19 medication, especially in high-risk groups.

## Interaction between ACE2 & gut microbiota in COVID-19 infection

There is a possible link between ACE2 expression, gut microbiota composition and COVID-19 severity. In addition to the protective catalytic activity of ACE2 in RAS, this enzyme plays critically RAS-independent roles in the GI tract, including the regulation of amino acids homeostasis, innate immunity and gut microbiota composition and the entrance receptor of SARS-CoV-2 [15]. Importantly, ACE2 regulates and stabilizes the expression of B0AT1, an apical membrane transporter in the small intestine, which mediates the uptake of neutral amino acids such as tryptophan [16]. In brief, tryptophan improves gut barrier function, reduces proinflammatory cytokines and induces the release of antimicrobial peptides, all of which help preserve the symbiotic gut microbiota composition. In the COVID-19 infection with ACE2 downregulation, the mentioned pathways are disrupted. In this situation, perturbed gut barrier function and disrupted microbiota community may promote cytokine storm in COVID-19 patients with severe clinical manifestation due to induced leaky gut and dysbiosis of gut microbiota. Consequently, the translocation of immunological components, such as lipopolysaccharide, from the GI lumen to the circulation increases and the proinflammatory mediators are abundantly produced [17].

Interestingly, a document reported the probable association between the gut microbiota members and ACE2 expression [18]. Moreover, a negative correlation was reported between ACE2 expression and *Bacteroides* spp. including *B. dorei, B. thetaiotaomicron, B. massiliensis* and *B. ovatus* in murine colonocytes, revealing the significance of the gut microbiota member in the regulation of ACE2 expression [19]. On the other hand, ACE2 upregulation is reported in dysbiosis correlated with unfavorable gut microbiota composition and increased inflammation. Accordingly, increased susceptibility to infection with SARS-CoV-2 can be caused by increased entrance of SARS-CoV-2 receptor, ACE2. This event could be explained by detecting SARS-CoV-2 RNA in the stool samples of many COVID-19 patients and GI clinical manifestations [20]. These documents demonstrated the crucial role of a symbiotic gut microbiota composition in determining the susceptibility and severity of COVID-19 using direct and indirect pathways, including the modulation of the gut barrier function, immune responses and ACE2 expression.

Furthermore, the ACE inhibitors and AT1R blockers augment ACE2 expression, which controls hypertension and inflammation resulting from elevated Ang II in hypertensive and diabetic patients. These patients could be in high-risk groups susceptible to infection with SARS-CoV-2 due to higher ACE2 levels that facilitate virus entrance [21]. It has also been well documented that these high-risk groups have a dysbiotic gut microbiota, and this could be considered as another possible explanation for increased susceptibility to COVID-19 infection because of elevated ACE2 and viral entrance.

## Conclusion

Accordingly, ACE2 could have a dual function in COVID-19: increasing infection or dampening inflammation and organ failure [2]. It is necessary to consider the complicated SARS-CoV-2 pathogenesis, which mainly consists of viral entry and disease development, which could be affected by the ACE2 levels. Although ACE2 mediates the onset of SARS-CoV-2 pathogenesis, restoring ACE2 levels (which are downregulated by SARS-CoV-2) could lead to cytokine storm, lung failure and dysbiotic gut microbiota. Furthermore, symbiotic gut microbiota may determine the susceptibility and severity of COVID-19 infection using suggested mechanisms, such as shaping lung immunity and the expression and activity of ACE2. In conclusion, understanding the significance of gut microbiota composition and ACE2 function and their relationship in SARS-CoV-2 pathogenesis could contribute to the better management of the COVID-19 pandemic.

## Future perspective

According to the complexity of SARS-CoV-2 pathogenesis, we need to fully understand the correlation of determining factors with host susceptibility to infection with SARS-CoV-2 and COVID-19 severity. Gut microbiota and RAS components, especially ACE2, are among these influential factors which can affect the SARS-CoV-2 pathogenesis. As mentioned, there is an association between the members of gut microbiota and ACE2 expression. ACE2 has a dual function by mediating SARS-CoV-2 entrance to the host cells and counterbalancing the deleterious RAS overactivity. On the other hand, altering ACE2 normal function can change gut microbiota composition and metabolic activity which may be followed by a dysregulated crosstalk with the host, resulting in a poor COVID-19 outcome. Gut microbiota plays a pivotal role in health and disease condition, and various factors (age, gender, genetic, geography, diet, antibiotic) affect microbiota composition. Therefore, we should evaluate the causative/protective impacts of microbiota on SARS-CoV2 pathogenesis and ACE2 function in distinct populations. In addition, these influential factors must be investigated in different high-risk groups to achieve comprehensive knowledge, which could be beneficial in designing therapeutic strategies (based on the gut microbiota intervention) to control and overcome to COVID-19 pandemic.
